# Integrating Bacterial and Viral Water Quality Assessment to Predict Swimming-Associated Illness at a Freshwater Beach: A Cohort Study

**DOI:** 10.1371/journal.pone.0112029

**Published:** 2014-11-19

**Authors:** Jason W. Marion, Cheonghoon Lee, Chang Soo Lee, Qiuhong Wang, Stanley Lemeshow, Timothy J. Buckley, Linda J. Saif, Jiyoung Lee

**Affiliations:** 1 Division of Environmental Health Sciences, College of Public Health, The Ohio State University, Columbus, Ohio, United States of America; 2 Food Animal Health Research Program, Ohio Agricultural Research and Development Center, Department of Veterinary Preventive Medicine, The Ohio State University, Wooster, Ohio, United States of America; 3 Division of Biostatistics, College of Public Health, The Ohio State University, Columbus, Ohio, United States of America; 4 Department of Food Science and Technology, The Ohio State University, Columbus, Ohio, United States of America; NERC Centre for Ecology & Hydrology, United Kingdom

## Abstract

**Background & Objective:**

Recreational waters impacted by fecal contamination have been linked to gastrointestinal illness in swimmer populations. To date, few epidemiologic studies examine the risk for swimming-related illnesses based upon simultaneous exposure to more than one microbial surrogate (e.g. culturable *E. coli* densities, genetic markers). We addressed this research gap by investigating the association between swimming-related illness frequency and water quality determined from multiple bacterial and viral genetic markers.

**Methods:**

Viral and bacterial genetic marker densities were determined from beach water samples collected over 23 weekend days and were quantified using quantitative polymerase chain reaction (qPCR). These genetic marker data were paired with previously determined human exposure data gathered as part of a cohort study carried out among beach users at East Fork Lake in Ohio, USA in 2009. Using previously unavailable genetic marker data in logistic regression models, single- and multi-marker/multi-water quality indicator approaches for predicting swimming-related illness were evaluated for associations with swimming-associated gastrointestinal illness.

**Results:**

Data pertaining to genetic marker exposure and 8- or 9-day health outcomes were available for a total of 600 healthy susceptible swimmers, and with this population we observed a significant positive association between human adenovirus (HAdV) exposure and diarrhea (odds ratio  = 1.6; 95% confidence interval: 1.1–2.3) as well as gastrointestinal illness (OR  = 1.5; 95% CI: 1.0–2.2) upon adjusting for culturable *E. coli* densities in multivariable models. No significant associations between bacterial genetic markers and swimming-associated illness were observed.

**Conclusions:**

This study provides evidence that a combined measure of recreational water quality that simultaneously considers both bacterial and viral densities, particularly HAdV, may improve prediction of disease risk than a measure of a single agent in a beach environment likely influenced by nonpoint source human fecal contamination.

## Introduction

Beach water advisories are issued to discourage human contact (e.g., swimming, wading, etc.) when water is potentially harmful to human health. In the United States, many of these advisories are issued at freshwater beaches when densities of fecal indicator bacteria (*E. coli* or enterococci) are observed or predicted to be in excess of single-day maximum criteria. Prior to November 26, 2012, the U.S. Environmental Protection Agency (USEPA) had established single-day maximum criteria, which were derived from several large epidemiological studies performed in the early 1980s [1]. In 2003, the 1986 *E. coli* criteria were supported by a comprehensive meta-analysis demonstrating *E. coli* was the most consistent indicator for predicting gastrointestinal illness in freshwater [2]. This same study indicated that methods for quantifying *E. coli* were problematic for same-day water quality advisories. The method required 18–24 h incubation and therefore was untimely for communication of risk to susceptible beachgoers [2].

Since the 2003 meta-analysis, several epidemiological studies evaluating associations between illness and more rapid measures of fecal indicators have been performed with an emphasis on genetic markers of bacterial or viral contamination, which have demonstrated the effectiveness of rapidly measured *Enterococcus* via quantitative polymerase chain reaction (qPCR) for predicting human illness at sewage-impacted Great Lakes and marine beaches [3], [4]. However, in epidemiology studies on swimmers at non-point source-impacted beaches, associations between qPCR-based bacterial markers and gastrointestinal (GI) illness were not observed [5], [6]. A potential reason for the lack of association in both studies could have related to the source of fecal contamination, which was likely dominated by avian sources [7], potentially presenting less risk for swimming-associated gastrointestinal (GI) illness than equivalent amounts of human-associated fecal contamination [8]. The diffuse nature of the source(s) of fecal contamination has been proposed as a possible explanation for the lack of association between bacterial genetic markers and human illness [9]–[11].

To date, few epidemiological studies on beachgoers have been performed at areas dominated by human-associated non-point source contamination, particularly in freshwater environments. Several peer-reviewed epidemiological studies have evaluated illness associations with human enteric viruses in European recreational waters [12]–[14] and U.S. waters [6], [15], [16]. Epidemiological studies pertaining to viruses are highly relevant as viruses are known to have a broad distribution in the aquatic environment [17], and are the most observed etiological agents in disease outbreaks associated with untreated U.S. recreational waters [18]. Recent epidemiological studies on swimmers [9]–[11] have suggested that viruses were the primary etiologic agents associated with recreational water illnesses based upon relatively short incubation periods following swimming exposure [10], [19]. Similarly with regards to incubation periods, among limited-contact recreational users of freshwater in Chicago, Illinois (U.S.A.), most gastrointestinal illnesses generally developed within three days of water exposure [19].

To date, the majority of recreational water-related epidemiological studies have been performed to identify effective single indicators, often bacterial, for practical application among those making beach management decisions. Multiple fecal indicators and multiple genetic markers have been evaluated in recreational waters with respect to their association with each other as well as GI illness frequency among swimmers. There is some inconsistency in the findings of studies that have studied associations (or the lack thereof) between fecal indicator densities and measured pathogen and/or viral levels in freshwater [20] and coastal nvironments [21], [22], and legitimate concerns remain regarding the use of single bacterial indicators for predicting and communicating swimming-associated illness risks to the general public [23]–[25].

To address these knowledge gaps, this study examined previously undescribed samples which were archived during our prospective cohort study [26] that gathered culture-based *E. coli* and human exposure data. In our 2010 study [26], new GI illnesses among swimmers were associated with increasing culture-based *E. coli* densities at East Fork Lake (Ohio, USA); however, no assessment of associations between genetic marker densities and human health occurred. In 2012, the U.S. Environmental Protection Agency (EPA) emphasized a need for additional evaluation of rapid molecular methods for the timely determination of water quality to protect recreational water users [27]. Building upon this need, we examined associations between GI illness incidence among swimmers exposed to multiple genetic markers targeting both viruses and bacteria in this non-point source human-impacted beach environment. This study presented a unique opportunity to successfully use our historical exposure and health data coupled with our previously unexplored genetic material to do a timely assessment of the effectiveness of viral and bacterial molecular markers for predicting GI illness risk among swimmers who used the East Fork beach in 2009.

## Materials and Methods

### Overall Approach

The exposure and health data were collected using the prospective cohort study approach adopted by Wade et al. in their epidemiological studies related to recreational water [3], [4]. This study builds upon our epidemiological investigations of recreational water-related illnesses by evaluating human illness associations with our recently qPCR-determined densities of bacteria and viruses. In brief, beach water samples, beach water quality data, and beach user exposure/behavior data were collected at East Fork State Park (39.0198° N; 84.1432° W) near Cincinnati, Ohio, U.S.A. Approvals to use this location for water sample collection and beachgoer interviews were kindly provided orally and in writing by Chris Dauner (Park Manager, East Fork State Park), and Dan West (Chief, Ohio Department of Natural Resources, Division of Parks and Recreation). Complete health and exposure data were collected from 891 beachgoers from 278 households. As described in our 2010 study [26], participants reported their health status via a telephone questionnaire after an eight- to nine-day follow-up period. More recently we quantified densities of four viral (human adenovirus (HAdV), human enterovirus (HEntV), and human norovirus genogroup I (HNoV GI) and genogroup II (HNoV GII)) and four bacterial (*E. coli* by *uidA* and 23S, Enterococci by 23S, *Bacteroides-Prevotella* by HuBac) markers from 23 samples collected over 23 weekend days from 2009 using qPCR and paired them with human exposure data.

### Ethics Statement

Approvals for the study design, questionnaires, verbal consent procedures, and related materials were obtained from the Institutional Review Board (IRB) at The Ohio State University (IRB Protocol #2009H0107). For this study, adult participants (18 years of age or older) capable of providing verbal consent were welcomed into the study by investigators or key personnel trained and authorized by the university to obtain consent. The written consent waiver permitting verbal consent was granted given the low study risk and because written consent would have required surname information and the gathering of consent documentation in a periodically windy and wet beach environment. Thus, written consent procedures would have increased the likelihood of losing or damaging documentation which would have then included participant surnames that could potentially be matched with telephone numbers and health data. Verbal consent was recorded on the datasheet for each household interviewed. In addition, participants were provided with a card sharing contact information for the principal investigator and the ethics coordinator in the Office of Responsible Research Practices at the university.

### Enrollment and Exposure Survey

The East Fork Beach Study [26] recruited beach user households into the study at the beach and interviewed them upon their departure to ascertain information regarding their activities at the beach on that same day. For each household in the study, an adult spokesperson provided investigators with information requested from a standard script. The obtained information was used for classifying exposure status and obtaining information related to potential confounding variables (age, gender, ethnicity, food consumption, source of food/drink, number of beach visits per year, duration of time at the beach, hand hygiene, extent of sand exposure, distance traveled, and number of other beach users). More specifically, individuals were asked whether any household members had: (1) no water contact; (2) waded, played or swam in the water; and/or (3) immersed their head in the water. Questions pertaining to any recent or on-going illnesses were also asked. Individual beach users were dichotomously classified as having or not having each of the above-mentioned exposures as well as any prevalent symptoms of gastrointestinal illness. These exposure data were recently paired with date-specific *E. coli* genetic marker density data to further establish our exposure classification of each beach user.

### Health Outcomes Survey

Health outcome data were obtained by Marion et al. [26] in which enrolled households were contacted eight to nine days after their beach visit via telephone. Only adult household members who were at the beach at the time of enrollment were eligible for interview participation. Interviews were conducted using a standard questionnaire inquiring about illness among household members at the beach at the time of enrollment. Reported illnesses were recorded as a yes/no response for a variety of symptoms (e.g., stomach cramps, nausea, diarrhea, vomiting, headache, fever, etc.) for each person who visited the beach. For comparing with other studies, persons were further classified by gastrointestinal illness. Similar to the Wade et al. studies [3], [4], persons were defined to have experienced “GI illness” if they were reported to have had any one of the following: nausea, stomach ache or stomach cramps, diarrhea (three or more loose or watery stools in a 24-hour period), or vomiting. With respect to nausea, all persons in this study who reported nausea were considered positive for GI illness. This GI illness definition is slightly different than the Wade et al. studies [3], [4], which only included nausea cases into their GI illness definition when the condition interfered with daily activities. In our study, we were unable to make this distinction as we did not ascertain if the nausea interfered with daily activities. Beyond the “GI Illness” definition, using the highly credible gastrointestinal illness (HCGI) definition employed by Colford [5] as “HCGI-1”, we likewise coded individuals as positive for HCGI if they were reported to have experienced any of the following: (1) vomiting; (2) diarrhea and fever, (3) stomach-ache and fever, or (4) nausea and fever.

### Sample Collection and Water Analysis

Beach water samples used for obtaining genetic marker data were collected on the same day as enrollment and administration of the exposure questionnaire on summer weekends during the time of day generally used for swimming. In total, 91% of samples were collected at the median time of 13:45±3 h. Two samples (9%) were collected after 16:00 on days when daily swimming attendance was low (n = 4, n = 28) in anticipation of more swimmers late in the day. For each day, 3 L was collected into multiple autoclaved 500 mL bottles (Nalgene, Rochester, NY, USA) and sterile Whirl-Pak bags (Nasco, Fort Atkinson, WI, USA) over 23 weekend days. Samples were collected near the beach center in water with an approximate depth of one meter by sweeping containers 30 cm below the water surface. Within 30 minutes after collection, a 1 L sample aliquot was used for membrane filtration for culturable *E. coli*, a 1 L sample aliquot was temporarily stored (24–48 h) at 4°C and then immediately filtered for bacterial marker analysis, and a 1 L aliquot was stored at −20°C for virus analysis.

*E. coli* densities were quantified for three separate filtration volumes (20, 50, and 100 mL) for each sample using the culture-based method described in EPA Method 1603 [27] and Marion et al. [26]. Other water quality parameters including temperature, pH, dissolved oxygen, and turbidity were also measured using a YSI 600XL data sonde (Yellow Springs Instruments, Yellow Springs, Ohio, U.S.A.) and a Hach 2100P Turbidimeter (Loveland, Colorado, U.S.A.) as described in Marion et al. [26]. UV index data were obtained from the National Oceanic and Atmospheric Administration [28]. Rainfall, lake stage (surface elevation), and lake inflow data were obtained from the U.S. Army Corps of Engineers [29].

A detailed description of sample preparation, water concentration, and quantification of the bacterial and viral marker densities by qPCR is provided as (**Text S1**), including a description of the primers and probes used in this study (**Table S1**). In brief, samples were filtered to capture bacteria for quantifying the following bacterial markers: *uid*A genes (uidA) and 23S rRNA genes (23S *E. coli*) of *E. coli*, 16S rRNA genes of *Bacteroides*-*Prevotella* (HuBac), and 23S rRNA genes of *Enterococcus* spp. (23S *Enterococcus*). For concentrating viruses (HEntV, HAdV, HNoV GI, and HNoV GII), membrane filtration was performed using cation (Al^3+^)-coated membranes. DNA or RNA was then extracted from the microbes captured on the membranes using a QIAmp DNA Stool Kit or the RNeasy Mini Kit (Qiagen, Valencia, CA, USA), respectively. RNA extracts for HEntV were amplified by reverse transcription (RT)-PCR and quantified using TaqMan real-time RT-PCR (RT-qPCR) with the QIAGEN OneStep RT-PCR Kit (Qiagen, Valencia, CA, USA). DNA extracts for HAdV and all bacterial markers were amplified by PCR and quantified using a TaqMan-based real-time qPCR system. All RT-qPCR and qPCR reactions except HNoV GI and HNoV GII were carried out in an ABI 48-well StepOne Real Time System (Applied Biosystems, Foster City, CA). RT-qPCR assays for HNoV GI and HNoV GII were carried out with an Eppendorf Mastercycler RealPlex^2^ (Eppendorf, Germany). Quantification of the markers was then determined using standard curves generated by plotting Ct values (y) versus the log gene copy numbers of the target microbes (x) and each limit of detection (LOD) was determined based on the lowest gene copy number from which above 90% of the replicates could be amplified in each qPCR assay. Additionally, with regards to these samples, the presence/absence of qPCR inhibition was previously evaluated for East Fork samples [20] using the Sketa22 qPCR assay as described in Haugland et al. [30].

### Data Analysis

The analysis focused on healthy susceptible individuals, and persons who were classified as prevalent or existing cases based upon responses to the enrollment questionnaire were excluded in analyses using incident cases. Logistic regression was employed to evaluate crude associations between genetic marker exposure and new illness among swimmers reporting head immersion. The data for the microbiological terms, including *E. coli* density data, were skewed, accordingly they were log (base 10)-transformed. Prior to log-transforming *E. coli* results, samples with 0 CFU/100 mL were assigned a value of 1 CFU/100 mL. In scatterplots and models containing genetic marker densities, for markers not detected after 45 cycles, densities were assigned a value of half the detection limit in log CFUs per 100 mL or log gene equivalents (GE) per 100 mL for bacterial and viral markers, respectively.

Multivariable logistic regression was used for estimating odds ratios for any GI illness, diarrhea, and HCGI among swimmers and non-swimmers (negative controls) as recommended in observational studies [31] and done by Colford et al. [9] in their beach user health study. The swimmer population was defined as beach users who reported immersing their head in the beach water. Non-swimmers were defined as beach users reporting no water contact or limited water contact that did not include head immersion in the beach water. All swimmers were assumed to have been exposed to the same density of *E. coli* and the various genetic markers observed from the single sample collection for that day. Densities of *E. coli* and the various genetic markers were determined from the single mid-day water sample collection.

The multivariable logistic regression models considered potential confounders and/or modifying influences, including covariates related to demographic, exposure, meteorological, and water quality factors. Since data were obtained from households, for the purpose of statistical analysis, the data were treated as clustered by household [32]. For constructing multivariable models, a backward selection approach was employed whereby covariates were dropped stepwise based on the highest p-value until arriving at the most parsimonious model [32]. The rule of ten events (cases) per covariate was used in logistic regression modeling [33]. Due to the low frequency of HCGI cases, HCGI model construction was limited to only two microbiological terms by relaxing the rule of ten events per covariate [34].

Multivariable logistic regression models were evaluated for fit via the Hosmer-Lemeshow Goodness-of-Fit Test [32] and for model discrimination via the area under the receiver-operating-characteristic curve (AUC) [32]. The modeling efforts and assessments of logistic model fit and discrimination were performed using Stata 11 (Stata Corporation, College Station, TX, USA). A time-series plot was generated with Minitab 16 (Minitab Inc., State College, PA, USA).

## Results

### Water Quality

**Figure 1:**
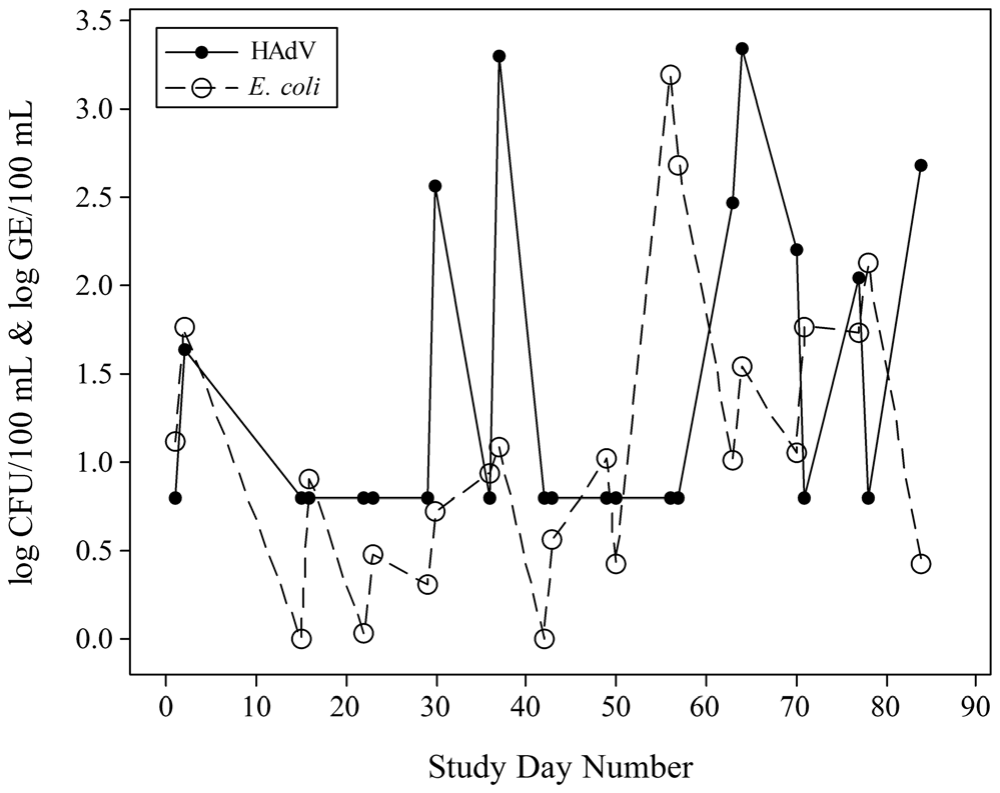
Time series plot for HAdV and *E. coli* densities measured during the 2009 swimming season at the study beach (East Fork Lake, Ohio).

Among the 23 weekend water samples collected for qPCR analysis, bacterial markers were more readily detected than viruses (Table 1). HEntV and HAdV were detected in 22% (5/23) and 35% (8/23) of all samples, respectively, whereas, HNoV GI and GII were not detected (0/23). As previously reported [20], qPCR inhibition was not observed in any of the 23 samples. Culturable *E. coli* densities greater than 0 CFU/100 mL were observed in 91% (21/23) of all samples. The time-series plot of culturable *E. coli* and HAdV densities by day (Figure 1) demonstrate the lack of association between the two water quality parameters, which was confirmed by correlation analysis (Spearman's ρ = 0.147; *p* = 0.503). Overall, culturable *E. coli* densities exceeded Ohio single-day maximum criteria (>235 CFU/100 mL) in 8.6% (2/23) of our samples; however, no beach advisories were issued by local or state authorities due to their adherence to the one sample per week guideline used by the Ohio Department of Health for inland waters. Other water quality parameters are summarized in Marion et al. [26].

**Table 1:** Microbial water quality measured by various genetic markers and *E. coli* at East Fork Lake, Ohio (N = 23).

Genetic Marker or Fecal Indicator	No. Samples Above LOD^a^	Detection Limit	Median	Range
HAdV (log gene equivalents/100 mL)	8	1.6	BD^b^	BD-3.34
HEntV (log gene equivalents/100 mL)	5	1.9	BD	BD-2.13
uidA (log copies/100 mL)	19	1.5	2.08	1.50–2.49
23S *E. coli* (log copies/100 mL)	19	2.46	2.94	2.46–3.19
HuBac (log copies/100 mL)	18	2.05	3.04	2.05–3.39
23S *Enterococcus* (log copies/100 mL)	17	1.93	2.19	1.93–2.88
*E. coli* (log CFU/100 mL)	21	ND^c^	1.01	LOD-3.19

a Limit of Detection or 0 CFU/100 mL for *E. coli*.

b Below Detection.

c Not Determined.

### Study Population

A total of 891 individuals were included in this study (from 278 households). The study population was 93% white, 2.5% black, 1% Hispanic and 3.5% other. The average study participant age was 24 years (median  = 21 years). Young children (≤5 years) represented 13% of the participants on whom data were available. Females were more represented (56%) than males (44%). The population described in this report is a subset of the 965 individuals from 300 households in our previous study [26], which was conducted over 26 weekend days. Genetic marker data were only obtained for 23 of 26 days of the 2009 East Fork Beach Study [26], and accordingly, this study reports the results of the investigation using genetic marker data. The collection of sufficient water for obtaining qPCR data was an additional task added to the previous study on the third sample day.

Among the participants in this study, 618 (69%) individuals reported head immersion in East Fork beach waters. Prevalent GI illness was reported for 18 (2.9%) of these swimmers at enrollment (Table 2). Chronic GI problems were self-reported at enrollment in 9 (50%) of these 18 individuals. All 18 individuals with prevalent GI illness (including chronic GI problems) were excluded from the exposure-related illness models. The prevalence of fever, nausea, and stomach cramping among participants was not ascertained at enrollment.

**Table 2:** Health status among study participants at enrollment and follow-up.

Reported Health Outcomes	Swimmers (n = 618)		Non-Swimmers (n = 274)	
	Health Status at Beach Enrollment (No. (%))	Health Status at Follow-up^a^ (No. (%))	Health Status at Beach Enrollment (No. (%)	Health Status at Follow-up^a^ (No. (%))
No Reported GI Problems	600 (97)	562 (94)	259 (95)	251 (97)
Any GI Illness	18 (2.9)	38 (6.3)	15 (5.5)	8 (3.1)
Chronic GI Problems	9 (1.5)	ND^b^	8 (2.9)	ND^b^
Diarrhea	6 (0.97)	28 (4.6)	5 (1.8)	8 (3.1)
Fever	ND	10 (1.6)	ND	2 (0.73)
Stomach Cramps	ND	8 (1.2)	ND	8 (1.2)
Nausea + Other GI Illness	ND	7 (1.2)	ND	1 (0.36)
Nausea Only	ND	3 (0.50)	ND	0 (0.00)
Vomiting	6 (0.97)	10 (1.6)	3 (1.1)	1 (0.36)
HCGI	ND	13 (2.2)	267 (97)	1 (0.37)

a Health status at telephone follow-up excluding positive cases at enrollment.

Not determined as information was not collected by the questionnaire.

### Water Quality and Human Illness

The qPCR-based densities for bacteria and viruses do not show any significant associations (*p*<0.05) with HCGI, GI, and diarrhea in single marker models using univariable and multivariable logistic regression when treating marker densities as continuous terms or binary terms in models (Table 3). Among all the continuous terms, HAdV had the strongest association with HCGI (crude OR (cOR)  = 1.6; 95% CI 0.90–2.9) and diarrhea (adjusted OR (aOR)  = 1.5; 95% CI 0.98–2.4); however, these associations were not significant but were noteworthy (*p* = 0.105; *p* = 0.066, respectively). When evaluating the association of either HEntV or HAdV detection with HCGI, GI, and diarrhea incidence, no association was observed (*p*>0.05). The covariates used in the illness models are summarized in **Table S2** and **Table S3**, respectively. The most significant covariate, food consumption at the beach (see **Table S2** and **Table S3**), was used in all genetic marker and *E. coli* models except the HCGI model, as food consumption at the beach was common to all HCGI cases. Beyond food consumption at the beach, specific conductivity and previous 72-hour UV results were the most significant covariates in all of the GI illness models. With respect to the diarrhea illness model, food consumption and previous 72-hour UV data were used as covariates, as they were identified as most significant through the backward elimination approach. In evaluating HCGI models relying on two microbiological terms, the only two-term model achieving significance was a combined model using HAdV and culturable *E. coli* (*p* = 0.003; likelihood ratio test). None of the bacterial markers were significant predictors of HCGI in models using an additional marker.

**Table 3:** Odds ratios for HCGI, GI, and diarrhea associated with exposure to varying levels of various molecular genetic markers and/or fecal indicators at East Fork Lake, Ohio.

Molecular Marker	HCGI		Any GI Illness		Diarrhea	
	cOR^a^ (95% CI)	Wald (*p*)	aOR^b^ (95% CI)	Wald (*p*)	aOR (95% CI)	Wald (*p*)
HEntV (+)^c^	1.6 (0.34–7.6)	0.552	0.76 (0.21–2.7)	0.674	0.17 (0.02–1.3)	0.081
HAdV (+)	2.1 (0.65–7.1)	0.212	1.3 (0.45–3.5)	0.654	2.2 (0.65–7.6)	0.202
uidA *E. coli* (+)	1.1 (0.22–5.4)	0.892	0.78 (0.27–2.2)	0.637	0.88 (0.24–3.2)	0.843
23S *E. coli* (+)	0.69 (0.18–2.7)	0.595	0.78 (0.30–2.0)	0.599	1.1 (0.31–3.8)	0.905
HuBac (+)	1.5 (0.32–7.1)	0.604	1.1 (0.38–3.2)	0.846	0.86 (0.25–2.9)	0.815
23S *Enterococcus* (+)	0.70 (0.21–2.4)	0.586	0.77 (0.27–2.2)	0.613	0.58 (0.18–1.9)	0.363
Log HAdV	1.6 (0.90–2.9)	0.105	1.2 (0.77–1.9)	0.399	1.5 (0.97–2.4)	0.066
Log uidA *E. coli*	1.0 (0.38–2.9)	0.927	0.76 (0.38–1.5)	0.442	0.98 (0.34–2.9)	0.974
Log 23S *E. coli*	0.78 (0.38–1.6)	0.489	0.85 (0.50–1.4)	0.526	1.2 (0.48–3.2)	0.648
Log HuBac	1.5 (0.63–3.7)	0.355	1.1 (0.67–1.8)	0.7	0.78 (0.49–1.2)	0.289
Log 23S *Enterococcus*	0.96 (0.38–2.4)	0.921	0.89 (0.49–1.6)	0.714	0.67 (0.37–1.2)	0.207

a Crude odds ratio.

b Adjusted odds ratios, see Table S2 and S3, to see the covariates used for adjustment.

c (+), Positive detection by qPCR, binary term.

Using this same bacteria and virus approach for GI illness, we observed a similar association. Since more GI cases were detected than HCGI, the model was able to contain an additional two covariates. In this model, significant (*p*<0.05) or near significant odds ratios (*p* = 0.056) were observed for all covariates in this model (Table 4), including food consumption, specific conductivity, *E. coli*, and HAdV. Increases in water conductivity were associated with protective effects (aOR<1); whereas, food consumption, increasing HAdV, and increasing *E. coli* were identified as risk factors for GI (aOR>1). The use of two microbial terms was supported in model building as the removal of the HAdV term resulted in significantly lower log-likelihood values for HCGI, GI, and diarrheal models (*p*<0.05), and the two microbial terms had the low p-values throughout the entire backward selection procedure allowing their retention in the model. The final four-term model was evaluated using a negative control group (the non-swimmer group), and no significant associations were observed for any model terms, suggesting no day-effects, and implying a water quality-related association in our exposed group. Lastly, all possible models for predicting GI illness using a single bacterial marker in tandem with either culturable *E. coli* or viral marker levels were developed, and no bacterial markers were associated with illness.

**Table 4:** Multivariable logistic regression models for predicting new HCGI^a^, GI, and diarrhea among swimmers immersing their head and beachgoers not immersing their head in beach water (East Fork Lake, Ohio).

Model & Exposure	Covariate	*β*	SE*_β_*	Wald (*p*)	AOR (95% CI)
HCGI Swimmers	Log HAdV (gene equivalents/100 mL)	0.8191	0.2864	0.005	2.3 (1.3–3.9)
	Log *E. coli* (CFU/100 mL)	1.101	0.3571	0.002	3.0 (1.5–6.1)
	Constant Term	−7.003			
GI Swimmers	Log HAdV (gene equivalents/100 mL)	0.421	0.1978	0.034	1.5 (1.0–2.2)
	Log *E. coli* (CFU/100 mL)	0.6515	0.2473	0.009	1.9 (1.2–3.1)
	Consumed Food at the Beach	1.543	0.7019	0.029	4.7 (1.2–19)
	Specific Conductivity (µS)	−0.092	0.0477	0.056	0.91 (0.83–1.0)
	Constant Term	19.37			
GI Non-Swimmers	Log HAdV (gene equivalents/100 mL)	0.0788	0.5481	0.886	1.1 (0.37–3.2)
	Log *E. coli* (CFU/100 mL)	0.2675	0.3346	0.425	1.3 (0.67–2.5)
	Consumed Food at the Beach	0.4254	0.8889	0.633	1.5 (0.26–8.9)
	Specific Conductivity (µS)	0.04	0.0279	0.154	1.0 (0.98–1.1)
	Constant Term	−15.33			
Diarrhea Swimmers	Log HAdV (gene equivalents/100 mL)	0.453	0.1977	0.023	1.6 (1.1–2.3)
	Log *E. coli* (CFU/100 mL)	0.3275	0.2466	0.186	1.4 (0.85–2.3)
	Constant Term	−4.226			
Diarrhea Non-Swimmers	Log HAdV (gene equivalents/100 mL)	0.0034	0.4905	0.994	1.0 (0.38–2.6)
	Log *E. coli* (CFU/100 mL)	0.2309	0.336	0.493	1.3 (0.65–2.4)
	Constant Term	−3.735			

a A model could not be constructed for HCGI among non-swimmers due to the low sample size resulting in an insufficient number of HCGI cases for model development.

Models constructed for predicting diarrhea using *E. coli* and HAdV preliminarily indicate that HAdV is associated with diarrheal disease incidence as the aOR for HAdV was significant (*p* = 0.023) when adjusting *E. coli* density by including *E. coli* density in the model. Compared to the HCGI and GI models, the diarrheal model provided poor discrimination (AUC <0.70) with respect to the ability of the model to properly classify diarrhea cases (Table 5). The Goodness-of-Fit test results (Table 5) also demonstrate the GI and HCGI models have better fits, as the p-values for those models are higher, indicating that the modeled results are not significantly different than the observed GI and HCGI results from the health survey.

**Table 5:** Model diagnostics for models from Table 4, pertaining to discrimination (AUC) and model calibration (goodness-of-fit).

Model Name for Swimmers	AUC^a^	Goodness-of-Fit^b^ (*p*)
Multivariable HCGI Model	0.75	0.895
Multivariable GI Model	0.753	0.958
Multivariable Diarrhea Model	0.64	0.206

a Area under the receiver-operator-characteristic curve (AUC).

b Hosmer-Lemeshow Goodness-of-Fit Test [32].

For considering possible interaction effects, a water quality index was constructed based upon the detection limit of HAdV and the median *E. coli* density. Table 6 presents the four water quality index values, and the respective *E. coli* and HAdV densities represented by each water quality index level. When evaluating associations for this model, the reference was set as the group of days in which *E. coli* densities were lowest and HAdV was not detected. Here we observe multiple associations with increasing viral and/or bacteria densities and both GI and diarrheal illness incidence. Swimmers with the presumed greatest exposure to *E. coli* and HAdV had the greatest odds of reporting new GI illness (OR  = 6.1; 95% CI 1.5–25) compared to swimmers with the least exposure to *E. coli* and HAdV. Exposure to the highest levels of *E. coli* when HAdV levels were low also presented increased GI illness risk (OR  = 5.4; 95% CI 1.5–19) compared to those swimmers with low *E. coli* and low HAdV exposure. In the diarrheal illness model, significantly increased odds of diarrhea were only observed in the group who swam on days with elevated *E. coli* and HAdV compared to the reference group (OR  = 5.2; 1.3–22). Model discrimination for each model (GI and diarrhea) was evaluated and the AUC values were 0.63 and 0.65, respectively.

**Table 6:** Odds ratios for GI and diarrhea associated with exposure to varying levels of HAdV and *E. coli* at East Fork Lake, Ohio.

Water Quality Index	Any GI Illness			Diarrhea		
	Cases/*n* (%)	OR (95% CI)	Wald (*p*)	Cases/*n* (%)	OR (95% CI)	Wald(*p*)
Group 1: Low HAdV & Low *E. coli* (HAdV <DL^a^, log *E. coli* <1.05)	3/187 (1.6)	Referent		3/189 (1.6)	Referent	
Group 2: Low HAdV & High *E. coli* (HAdV <DL, log *E. coli*>1.05)	16/199 (8.0)	**5.4 (1.5–19)**	**0.011**	9/199 (4.5)	2.9 (0.74–12)	0.126
Group 3: High HAdV & Low *E. coli* (HAdV> DL, log *E. coli* <1.05)	11/126 (8.7)	5.9 (0.88–39)	0.067	9/127 (7.1)	4.7 (0.56–40)	0.153
Group 4: High HAdV & High *E. coli* (HAdV> DL, log *E. coli*>1.05)	8/88 (9.0)	**6.1 (1.5–25)**	**0.011**	7/90 (7.8)	**5.2 (1.3–22)**	**0.023**

a Detection Limit.

## Discussion

This prospective cohort study from an inland U.S. beach demonstrates the predictive potential of an integrative, multi-microbial approach for estimating recreational waterborne disease risk from viral and bacterial indicators. The term ‘indicator’ used here does not imply fecal indicator, but instead refers to ‘health-relevant’ indicators. Our results showed a positive association between increasing densities of individual health-relevant indicators (HAdV and *E. coli*) and increased odds of GI and HCGI among swimmers at the studied beach. More importantly, the two-indicator model (HAdV and *E. coli*) represented a significant improvement over single health indicator approaches with this data. Although speculative, particularly due to the small sample size of the study, the findings of this two-indicator approach are etiologically plausible as a combined viral + bacterial (ViBac) approach may account for GI and HCGI illness associations with viral and bacterial pathogens. Based upon our cohort study results, we have some reason to speculate that a multiple health-indicator approach may be beneficial for natural recreational waters such as the East Fork beach, which is part of a watershed comprised of mixed land uses and a high density of septic systems, all of which may promote a more diffuse type of fecal contamination than observed in point-source impacted waters. Within the beach watershed of interest, two municipal wastewater treatment plants (Williamsburg and Bethel WWTPs) and several smaller WWTPs operate, while malfunctioning septic systems are presumed to be a very significant source of contamination [35]. In the East Fork of the Little Miami River watershed, which includes the study beach, the septic system density in sub-catchments has been directly linked to human-associated fecal contamination through the use of human-associated genetic markers [35]. The only Harsha Lake (East Fork Lake) tributary investigated by Peed et al. [35] had the highest mean human-specific marker (HF183) density and the second highest density of septic systems (41/km^2^) among tributaries investigated suggesting diffuse septic system-associated fecal contamination impacts this beach.

In health studies of swimmers in waters impacted by nonpoint source and/or diffuse fecal contamination, the lack of association between bacteria-based genetic markers and human illness has been observed [5], [6], [10], [16]. Similarly, we did not observe associations between qPCR-based bacterial densities and human illness at our inland freshwater beach. The association of culturable *E. coli* with HCGI and GI was already observed [26], but the lack of association of GI and HCGI with human-specific genetic markers (e.g., HuBac) was unknown in this study population. Two possible reasons for our findings are as follows: (1) transport and fate varies considerably between bacterial genetic markers and viable waterborne pathogens, including viruses; and/or (2) our sample size was not big enough to detect significant associations.

With respect to viruses at the beaches with diffuse contamination, male-specific coliphage density has been associated with HCGI and GI illness in marine studies [5], [16]. Similarly, our study observed an association between a virus (HAdV) and GI illness; however, unlike the marine studies, our study did not evaluate coliphages, focusing solely on viral pathogens. Like the associations between coliphages and illness [5], [16], our finding of HAdV associations with HCGI and GI incidence in the adjusted models [Table 4] preliminarily suggests a possible benefit of using a viral indicator for evaluating recreational water illness risk in certain environments.

Similar to the Colford et al. study [5], human noroviruses (HNoV GI and HNoV GII) were not detected; however, human adenoviruses (HAdV) were detected from eight (35%) samples at East Fork versus one detection in the Colford et al. study [5]. The infrequent detection of HEntV (22%) in our study is not entirely unexpected as enteroviruses have been observed at densities below 1 PFU/100 mL in European waters [36] and have been infrequently detected (9% of samples) elsewhere [37]. These findings are similar to earlier findings from Fleisher et al. [14] that determined positive enterovirus detection (by cell culture) was less likely than negative detection in United Kingdom waters, which was also described as a limitation of the utility of the enterovirus assay for swimming advisory determination. The low detection frequency of viruses coupled with practicality of measurement presents challenges for establishing their use unless monitored in tandem with other indicators of water quality. Their use in a monitoring scheme may complement fecal indicator bacteria which have not effectively predicted viral densities in freshwater and marine environments [17], [21], [38], [39]. Among viruses, adenovirus has shown promise as a potential human-associated marker of fecal contamination, whereby adenovirus presence has been linked to human-associated fecal problems as reflected by human-associated bacterial markers [40], [41].

Despite a lack of association between viruses and bacteria, swimming advisories are primarily bacteria-based for practical reasons. Furthermore, there appears to be growing consensus among experts suggesting viruses are responsible for many water-related illnesses [18], [19], [42]. It is becoming more widely understood that enteric viruses (e.g., noroviruses, rotaviruses) are responsible for a substantial number of GI illnesses among U.S. beach users [18]. With viruses gaining attention and being more frequently measured, approaches for monitoring viruses to support integrative health risk models for water-associated illnesses are meaningful. Viral indicators of GI illness, particularly enteroviruses and bacteriophages, were described as promising predictors in 2003 by Wade et al. [2], but were also described as being limited by the difficulty and time for cultivation and enumeration. Now, with increasing use of molecular approaches such as qPCR, viral detection and enumeration is becoming less difficult and more rapid. In less contaminated or nonpoint source impacted waters, the use of a more commonly detected virus, like HAdV, may serve as an effective viral marker over noroviruses or enteroviruses for beach monitoring, since the detection frequency of adenovirus (36%) has been observed to be much greater than norovirus detection (9%) in European water samples [41], [43].

The low detection frequency of norovirus in our samples and elsewhere may involve RNA virus instability, unstable DNA amplification potential from single-stranded RNA, and virus seasonality since the most prevalent human norovirus (GII.4) has clear winter peak seasonality [44] and therefore is less likely to be observed in summer surface waters. Unlike HNoV and HEntV, HAdV is a double-stranded DNA virus presumed to have better stability in environments and greater opportunities for successful detects. Furthermore, adenoviruses are believed to be up to 60 times more resistant to damage from ultraviolet irradiation than RNA viruses [45] likely affording HAdV particles greater integrity and viability leading to more undamaged infectious particles [46]. Given detection frequencies, Wyn-Jones et al. [41] encourages the consideration of adenovirus over norovirus as a recreational water quality indicator

Accordingly, water quality advisories should be protective for preventing human illness from all potential infectious agents as deemed practical with the available methods. The need for bacteria-based indicators of health risk are warranted, as the historical paradigm supports the notion that individual fecal indicators like *E. coli* and enterococci as well as human-specific markers explain the potential pathogenic bacterial genera (*Shigella, Camplyobacter*, *Salmonella*, etc.) as well as gastrointestinal illness among swimmers. However, the need for data attempting to address and/or quantify viral-associated human health risks is significant, and some attempt has recently been made using a site-specific quantitative microbial risk assessment (QMRA) for adenovirus illness [47]. Although speculative, virus monitoring may be beneficial in recreational waters where the contamination sources of the aquatic system are complex and impacted by a combination of non-point source human-associated contamination as well as agricultural runoff, as in Kundu et al. [47]. The concept of ‘know your beach’ appears to be particularly important as it relates to water quality when fecal indicator bacteria are low, and some consideration to broadening the paradigm to include multiple microbiological terms may be warranted with further research, with a particular emphasis on viruses capable of predicting recreational water illnesses more efficiently. Similar approaches demonstrating the value of a holistic design, like our combined virus-bacteria model, for enhancing risk assessments pertaining to water are already gaining attention in microbial source tracking studies using toolbox approaches [22]. Beyond water quality analysis, additional assessments of other known exposures presenting health risks, such as sand quality [48] and hydrology [49] are also recommended to be considered for more holistic beach condition modeling [25]. In this study, our integrative risk model using two microbiological terms, a fecal indicator bacteria and a viral genetic marker, provided a better assessment of human health risk than any single indicator/marker approach with limited data. Additional epidemiological studies using HAdV or other indicators of viral contamination that control for illnesses explained by bacteria are warranted for improving our predictive capability of swimming-related illness as well as our characterization of water quality at inland beaches.

### Study Limitations

Future studies with larger populations are needed for drawing less speculative conclusions. The total swimmer population after exclusion is relatively small (n = 600) for rigorous analysis and may be leading us to spurious conclusions. Given the low number of cases presented, model construction was limited to two to four covariates, which therefore limited our ability to adequately assess multiple confounders, interaction effects, and linearity. The cohort study approach used here and in other U.S. studies [1], [3], [4] also presents several methodological limitations [50] related to potential misclassification of exposure and disease among study participants. We acknowledge a variety of confounders (assessed and not assessed) may be responsible for GI illness cases beyond adverse water quality conditions, such as food consumption at the beach. For example, personal risk perceptions were not measured in this study, which have been linked to self-reported illness among swimmers in other studies [15]. To reduce self-selection bias among swimmers, this study performed modeling comparing swimmers in various exposure groups to a low exposure swimmer group. Exposure misclassification is also relevant, particularly if beach water quality varies significantly throughout each study day which would limit reliable exposure estimates from a single daily sampling effort. Lastly, illness misclassification relying on self-reported information was more likely in this study than in studies using direct follow-up examinations.

Despite this sample size limitation, several constructed models did provide acceptable discrimination (AUC>0.70; Table 5) and acceptable model fit, particularly the HCGI and GI models from Table 4. Although the study presents several limitations, the study likely represents the only prospective epidemiological investigation exploring HAdV associations with recreational water-associated illness at this important beach type (inland lake recreational water). Future studies with larger samples sizes are warranted, particularly in beach environments where fecal contamination is human-associated and from dispersed sources or a mixture of point and dispersed sources.

## Conclusions

- No association was observed between exposure to various qPCR-measured bacterial markers and recreational water-associated illness among study participants.
- Increasing levels of qPCR-measured HAdV were associated with increased odds of recreational water-associated gastrointestinal illness among the swimmers.
- Combined measurement of HAdV and culturable *E. coli* densities potentially enhances recreational water illness prediction among swimmers.
- Future studies with larger sample sizes enabling adjustment for additional confounding variables are needed to permit more robust investigations of human illness associations with exposures to HAdV and other viral markers in recreational water environments.

## Supporting Information

Table S1 **Primers and probes used in this study.**
(DOCX)

Table S2 **Adjusted odds ratios for covariates used in GI illness models from** **Table 3** **.**
(DOCX)

Table S3 **Adjusted odds ratios for covariates used in diarrheal illness models from** **Table 3** **.**
(DOCX)

Text S1 **Detailed methods for sample processing and qPCR procedures.**
(DOCX)
