# Supplementary material for: Integrating Bacterial and Viral Water Quality Assessment to Predict Swimming-Associated Illness at a Freshwater Beach: A Cohort Study
Source: PLoS One. 2014 Nov 19;9(11):e112029. doi: 10.1371/journal.pone.0112029 (PMC4237328; doi:10.1371/journal.pone.0112029)
Supplement: Table S1 — Primers and probes used in this study. (DOCX) [file pone.0112029.s001.docx]

**Table S1.** Primers and probes used in this study.

| Assay name | Primer or probe | Sequence (5’-3’)^a^ | Amplicon size (bp) | Gene | Annealing and extension | Target(s) | Reference(s) |
| --- | --- | --- | --- | --- | --- | --- | --- |
| HAdV | AQ1 | GCCACGGTGGGGTTTCTAAACTT | 131 | Hexon gene | 55°C, 15 s for annealing and 65°C, 1 min for extension | human adenovirus | [51] |
|  | AP | FAM-TGCACCAGACCCGGGCTCAGGTACTCCGA-MGB^a^ |  |  |  |  |  |
|  | AQ2 | GCCCCAGTGGTCTTACATGCACATC |  |  |  |  |  |
| HEntV | EV1 | GATTGTCACCATAAGCAGC | 147 | 5'-untranslated region | 60°C, 1 min | Human enterovirus | [49] |
|  | EV-PROBE | FAM-CGGAACCGACTACTTTGGGTGTCCGT-MGB |  |  |  |  |  |
|  | EV2 | CCCCTGAATGCGGCTAATC |  |  |  |  |  |
| HNoV GI | COG1F | CGYTGGATGCGNTTYCATGA^b^ | 84 | ORF1-ORF2 junction region | 56°C, 1 min | Human norovirus genogroup I | [50] |
|  | COG1R | CTTAGACGCCATCATCATTYAC |  |  |  |  |  |
|  | RING1(a)-TP | FAM-AGATYGCGATCYCCTGTCCA-BHQ^c^ |  |  |  |  |  |
|  | RING1(b)-TP | FAM-AGATCGCGGTCTCCTGTCCA-BHQ |  |  |  |  |  |
| HNoV GII | COG2F | CARGARBCNATGTTYAGRTGGATGAG | 97 |  |  | Human norovirus genogroup II |  |
|  | COG2R | TCGACGCCATCTTCATTCACA |  |  |  |  |  |
|  | RING2-TP | FAM-TGGGAGGGCGATCGCAATCT-MGB |  |  |  |  |  |
| uidA | UidAF | CAACGAACTGAACTGGCAGA | 130 | *uid*A | 60°C, 1 min | *E. coli* | [52] |
|  | UidAP | FAM-TTCTCTGTTGAAAGGCGCTT-MGB |  |  |  |  |  |
|  | UidAR | GCCGTTACCTCACCGTCTA |  |  |  |  |  |
| 23S *E. coli* | EC23S857F | GGTAGAGCACTGTTTTGGCA | 87 | 23S rRNA | 60°C, 1 min | *Enterococcus* spp. | [53] |
|  | EC23S857P | FAM-TCATCCCGACTTACCAACCCG-MGB |  |  |  |  |  |
|  | EC23S857R | TGTCTCCCGTGATAACTTTCTC |  |  |  |  |  |
| HuBac | qHS601F | GTTGTGAAAGTTTGCGGCTCA | 150 | 16S rRNA | 62°C, 1 min | *Bacteroides/*  *Prevotella* | [54, 55] |
|  | qHS624MGB | FAM-CGTAAAATTGCAGTTGA-MGB |  |  |  |  |  |
|  | qBac725R | CAATCGGAGTTCTTCGTGATATCTA |  |  |  |  |  |
| 23S *Enterococcus* | ECST784F | AGAAATTCCAAACGAACTTG | 91 | 23S rRNA | 60°C, 2 min | *Enterococcus* spp. | [56, 57] |
|  | GPL813TQ | FAM-TGGTTCTCTCCGAAATAGCTTTAGGGCTA-MGB |  |  |  |  |  |
|  | ENC854R | CAGTGCTCTACCTCCATCATT |  |  |  |  |  |

^a^FAM: 6-carboxyfluorescein; MGB: minor groove binder; BHQ: black hole quencher.

^b^Single-letter code: B stands for G, T, or C; N stands for A, C, G, or T; R stands for A or G; and Y stands for C or T.
