# Supplementary material for: Integrating Bacterial and Viral Water Quality Assessment to Predict Swimming-Associated Illness at a Freshwater Beach: A Cohort Study
Source: PLoS One. 2014 Nov 19;9(11):e112029. doi: 10.1371/journal.pone.0112029 (PMC4237328; doi:10.1371/journal.pone.0112029)
Supplement: Table S2 — Adjusted odds ratios for covariates used in GI illness models from Table 3 . (DOCX) [file pone.0112029.s002.docx]

**Table S2.** Adjusted odds ratios for covariates used in GI illness models from Table 3.

|  |  | | |
| --- | --- | --- | --- |
| Genetic Marker Term for GI Illness Models in Table 3. | **Covariates and Corresponding Adjusted Odds Ratios (95%CI)** | | |
|  | Consumed Food at the Beach | Specific  Conductivity (μS) | 72-hour UV Average |
| HEntV (+) | 5.8 (1.7-20) | 0.91 (0.84-0.97) | 0.73 (0.58-0.92) |
| HAdV (+) | 5.6 (1.7-18) | 0.91 (0.85-0.97) | 0.74 (0.60-0.91) |
| Log HAdV | 5.6 (1.6-19) | 0.91 (0.85-0.98) | 0.74 (0.59-0.92) |
| uidA *E. coli* | 6.1 (1.9-20) | 0.90 (0.83-0.97) | 0.73 (0.59-0.91) |
| 23S *E. coli* | 5.9 (1.7-20) | 0.90 (0.84-0.97) | 0.73 (0.59-0.90) |
| HuBac | 5.9 (1.7-20) | 0.90 (0.84-0.97) | 0.72 (0.58-0.91) |
| 23S *Enterococcus* | 5.8 (1.7-20) | 0.91 (0.84-0.98) | 0.74 (0.60-0.93) |
