# Supplementary material for: Integrating Bacterial and Viral Water Quality Assessment to Predict Swimming-Associated Illness at a Freshwater Beach: A Cohort Study
Source: PLoS One. 2014 Nov 19;9(11):e112029. doi: 10.1371/journal.pone.0112029 (PMC4237328; doi:10.1371/journal.pone.0112029)
Supplement: Table S3 — Adjusted odds ratios for covariates used in diarrheal illness models from Table 3 . (DOCX) [file pone.0112029.s003.docx]

**Table S3.** Adjusted odds ratios for covariates used in diarrheal illness models from Table 3.

| Genetic Marker Term for GI Illness Models in Table 3. | **Covariates and Corresponding Adjusted Odds Ratios (95%CI)** | |
| --- | --- | --- |
|  | Consumed Food at the Beach | 72-hour UV Average |
| HEntV (+) | 3.9 (0.99-16) | 0.85 (0.71-1.0) |
| HAdV (+) | 3.7 (0.89-16) | 0.84 (0.68-1.0) |
| Log HAdV | 3.8 (0.90-16) | 0.83 (0.68-1.0) |
| uidA *E. coli* | 3.7 (0.95-14) | 0.85 (0.72-1.0) |
| 23S *E. coli* | 3.7 (0.93-15) | 0.85 (0.72-1.0) |
| HuBac | 3.6 (0.91-14) | 0.86 (0.72-1.0) |
| 23S *Enterococcus* | 4.1 (0.37-1.2) | 0.86 (0.73-1.0) |
