# Supplementary material for: Integrating Bacterial and Viral Water Quality Assessment to Predict Swimming-Associated Illness at a Freshwater Beach: A Cohort Study
Source: PLoS One. 2014 Nov 19;9(11):e112029. doi: 10.1371/journal.pone.0112029 (PMC4237328; doi:10.1371/journal.pone.0112029)
Supplement: Text S1 — Detailed methods for sample processing and qPCR procedures. (DOCX) [file pone.0112029.s004.docx]

**Text S1. Detailed methods for sample processing and qPCR procedures.**

*Sample processing and nucleic acid extraction*

Water concentration for bacterial DNA extraction was performed using the procedure in Lee et al. [51]. Briefly, water samples (200-250 mL) were pre-filtered through a 20 μm pore size nylon filter membrane (Osmonics, Minnetonka, MN, USA) to remove algae and debris [52] and then filtered through a 0.45 μm pore size mixed cellulose ester filter membrane (Pall Corporation, Ann Arbor, MI, USA). The membrane was transferred into a 50 mL sterile tube and DNA was extracted using QIAamp^®^ DNA stool kit (Qiagen, Valencia, CA) according to the manufacturer’s instruction. The final eluates were used immediately or stored at -80°C until further processing.

Viruses were captured and concentrated from water samples using the cation-coated filter method [53]. Briefly, each water sample was filtered through a cation-coated cellulose membrane (0.45 μm pore size, 90 mm diameter; Millipore, Bedford, MA, USA) saturated with 5 mL of 250 mM AlCl_3_·6H_2_O (Acros Organics, Geel, Belgium), followed by eluting and concentrating viruses using Centriprep centrifugal filters (Centriprep^®^ YM-50 tube; Millipore, Bedford, MA, USA) as described previously [20]. The final concentrated solution containing viruses was used for viral DNA or RNA extraction using the QIAamp^®^ DNA Stool Kit or the RNeasy Mini Kit (Qiagen, Valencia, CA, USA) according to the manufacturer’s instructions, respectively.

*qPCR for quantification of genetic markers*

For HEntV and HNoV (genogroup I [GI]and genogroup II [GII]) quantification, one-step TaqMan^®^ reverse transcription (RT)-qPCR assays were performed using the QIAGEN^®^ OneStep RT-PCR Kit (Qiagen, Valencia, CA, USA) according to the manufacturer’s instruction. Briefly, a mixture for each qPCR assay was composed of 5 µL of 5-fold diluted RNA template, 400 nM each forward and reverse primer, 10 U of RNaseOUT™ Recombinant Ribonuclease Inhibitor (Invitrogen, Carlsbad, CA), and 0.1 µM ROX dye (6-carboxy-X-rhodamine; Stratagene, La Jolla, CA, USA) as a passive reference dye, and appropriate probe concentrations (200 nM for HEntV; 300 nM RING1(a)-TP and 100 nM RING1(b)-TP for HNoV GI; 100 nM RING2-TP for HNoV GII) described in the supplemental material (**Table S1**) as described previously [54, 55]. Thermal conditions included reverse transcription at 50°C for 30 min, heat inactivation of reverse transcriptase and initial activation of HotStart polymerase by incubation at 95°C for 15 min, and amplification for 45 cycles by denaturing at 95°C for 15 s followed by annealing and extension under proper conditions (**Table S1**). HAdV qPCR assay was performed in a similar way but for a use of the TaqMan^®^ universal PCR master mix (Applied Biosystems, Foster City, CA, USA) and appropriate primer and probe set (**Table S1**) as described previously [56]. The PCR protocol was composed of an initial cycle at 50°C for 2 min and 95°C for 10 min, followed by 45 cycles of denaturation at 95°C for 15 s, and annealing and extension under proper conditions (**Table S1**).

For the quantification of fecal bacterial markers, TaqMan^®^-based real-time qPCR analysis was performed in duplicate targeting *uid*A genes (uidA) and 23S rRNA genes (23S *E. coli*) of *E. coli*, 16S rRNA genes of *Bacteroides*-*Prevotella* (HuBac), and 23S rRNA genes of *Enterococcus* spp. (23S *Enterococcus*) as previously described by Chern et al. [57, 58], Bernhard and Field [59], Okabe et al. [60], Ludwig and Schleifer [61], and U.S. Environmental Protection Agency [62], respectively with minor modification. The qPCR mixture consisted of a total volume of 25 µL containing 5 µL of 5-fold diluted DNA template, 12.5 µL of TaqMan^®^ universal PCR master mix (Applied Biosystems, Foster City, CA), 400 nM of each primer set, and 200 nM of each probe (**Table S1**). The PCR protocol included an initial cycle at 50°C for 2 min and 95°C for 10 min, followed by 45 cycles of denaturation at 95°C for 15 s, and annealing and extension under proper conditions (**Table S1**). The concentrations of bacterial and viral markers except 23S *E. coli* and HNoV GI markers from each sample were determined using standard curves generated in our previous studies [20, 52, 63]. Standard curves 23S *E. coli* and HNoV GI markers were generated from serial dilutions of DNA of *E. coli* DH5α and RNA transcript of Hu/NoV/GI/Norwalk (GenBank accession number: M87661; generously provided by Dr. Mary K. Estes at Baylor College of Medicine), respectively, by plotting Ct values (y) versus gene copy number (23S *E. coli*) or gene equivalents (HNoV GI) (x). The positive controls used in this study were: human poliovirus 1 (strain LSc; ATCC VR-59), human adenovirus 41 (strain Tak; ATCC VR-930), Hu/NoV/GI/Norwalk (GenBank accession number: M87661), Hu/NoV/GII.4/HS194/2009/US (GenBank accession number: GU325839), *E. coli* DH5*α*, *Bacteroides fragilis* (ATCC 25285^T^), and *Enterococcus* *faecium* (ATCC 19434^T^). Sterile phosphate buffered saline (PBS) were used as negative controls in DNA and RNA extraction.
